# Assessment of hemoglobin mass and blood volumes at moderate altitude using carbon monoxide rebreathing method

**DOI:** 10.1097/MD.0000000000042762

**Published:** 2025-06-06

**Authors:** Husain Alkhaldy, Meshal M. Alqahtani, Mahdi S. Al Amri, Yusra D. Alasmari, Ramy Mohamed Ghazy, Mohammed Alshehri

**Affiliations:** a Department of Internal Medicine, College of Medicine, King Khalid University, Abha, Saudi Arabia; b College of Medicine, King Khalid University, Abha, Saudi Arabia; c Department of Intensive Care, King Khalid University Hospital, Abha, Saudi Arabia; d Department of Radiology, King Khalid University Hospital , Abha, Saudi Arabia; e Department of Family and Community Medicine, College of Medicine, King Khalid University, Abha, Saudi Arabia; f Tropical Health Department, High Institute of Public Health, Alexandria University, Alexandria, Egypt.

**Keywords:** carbon monoxide rebreathing method, hemoglobin, high-altitude, high-altitude, lean body mass normalization, red blood cell volume

## Abstract

At high altitudes, increased red blood cell volume (RBCV) and reduced plasma volume (PV) complicate interpretation of hemoglobin (Hb) and hematocrit (HCT) values. Whether moderate altitude affects these parameters remains debated. This study aimed to assess hemoglobin mass (Hb mass) and intravascular volumes at moderate altitude. Participants were recruited from a moderate-altitude population (2250 m above sea level) in the Aseer region, Saudi Arabia. Eligible individuals were healthy, with no medical conditions or prescribed medications. Participants with baseline Hb and HCT values outside the reference range were excluded. Hb mass, blood volume (BV), RBCV, and plasma volume (PV) were measured using the carbon monoxide (CO) rebreathing method. Body composition was assessed using dual-energy X-ray absorptiometry (DEXA). Results were reported as absolute values and normalized to body weight (BW) and lean body mass (LBM). Ninety-four participants (30 females, 64 males; mean age 25.8 ± 10.6 years) were included. Males had significantly higher absolute Hb mass (922.8 ± 195.0 vs 594.9 ± 91.2 g, *P* < .001), BV (5625.0 ± 1209.0 vs 4231.0 ± 881.0 mL, *P* < .001), PV (2903.0 ± 717.0 vs 2480.0 ± 552.0 mL, *P* = .005), and RBCV (2721.2 ± 56.9 vs 1749.8 ± 37.8 mL, *P* < .0001), as well as higher normalized Hb mass^(BW)^ (11.7 ± 3.1 vs 9.9 ± 2.4 g/kg^BW^, *P* = .007) and RBCV^BW^ (34.6 ± 8.8 vs 29.2 ± 7.1 mL/kg, *P* = .004). No gender differences were found in BV (*P* = .658) or PV (*P* = .155) when normalized to BW. Differences in Hb mass (*P* = .37) and RBCV (*P* = .39) also disappeared when normalized to LBM. However, females had higher BV^L^^BM^ (126.8 ± 23.7 vs 110.9 ± 15.3 mL/kg^LBM^, *P* = .007) and PV^LBM^ (75.0 ± 14.2 vs 56.6 ± 8.2 mL/kgLBM, *P* < .001). Compared to historical sea-level controls, altitude participants showed higher absolute Hb mass in males (*P* = .01) and females (*P* = .014), with higher values per LBM (*P* < .0001 for both). Hb and HCT showed significant but weak correlations with Hb mass and intravascular volumes. Residents at moderate altitudes have elevated Hb mass compared to sea-level references using the CO rebreathing technique. Population- and altitude-specific reference ranges for blood volumes and Hb mass CO rebreathing technique are needed for clinical application.

## 1. Introduction

The blood volume (BV), with its 2 main compartments of plasma volume (PV) and red blood cell volume (RBCV), is a controlled milieu that is subject to alteration by both physiological and pathological processes. In clinical practice, BV assessment usually relies on the simple, readily available, and straightforward measurements of hemoglobin (Hb) concentration and hematocrit (HCT), as a change in either RBCV or PV, or both, will affect these values. However, the corollary is that Hb can be affected by a change in PV without any change in RBCV and vice versa, or by the combination of changes in both.^[1]^ Thus, a contraction in PV could result in erroneous diagnosis of polycythemia, i.e. spurious polycythemia. Conversely, expansion of PV with heart failure and chronic kidney disease results in dilutional anemia, and only to a lesser degree in actual anemia.^[2]^

At high-altitudes, altitude-associated physiological adaptations can complicate the interpretation of Hb values. Specifically, PV decreases within the first hours at altitude, and RBCV increases within weeks of exposure.^[3,4]^ The increase in Hb that is observed with altitude is the result of these combined changes, but may not be uniform, i.e., within the first few days of altitude exposure, the contribution of RBCV changes to the altered Hb is absent but gradually increases with time spent at high-altitude. If a pathological process also occurs, it is impossible to delineate which process is responsible for the change in Hb. Furthermore, while increased hemoglobin mass (Hb mass) is associated with higher Hb concentration, the relationship is not linear; 2 individuals with the same Hb level may have different true Hb mass and PV values.^[1]^

The current criterion for the diagnosis of anemia is Hb below 12 g/dL for women and 13 g/dL for men.^[5]^ However, since this parameter is concentration-based and is known to be influenced by altitude exposure, the criteria for diagnosing anemia and polycythemia should be reevaluated in the case of high-altitude residency. In the absence of robust evidence, the influence of moderate altitude on RBCV and PV remains poorly understood, as are their respective contributions to increased Hb.

We hypothesized that moderate-altitude exposure results in an increase in RBCV and a decrease in PV, thereby necessitating reevaluation of the current sea-level-based Hb and HCT criteria used for diagnosing anemia and polycythemia for their utility in permanent altitude residents of Southern Saudi Arabia, a densely populated region situated at 2200 to 3000 m above sea level. Additionally, it is hypothesized that providing reference values for RBCV and PV at moderate altitudes will enhance the accuracy of diagnosing blood-related conditions in these environments. The current study aimed to provide reference values for RBCV and PV from a moderate-altitude population, i.e. an altitude of relevance to more than 1 million inhabitants of Saudi Arabia.

## 2. Methods

### 2.1. Study design and setting

**Figure 1.**
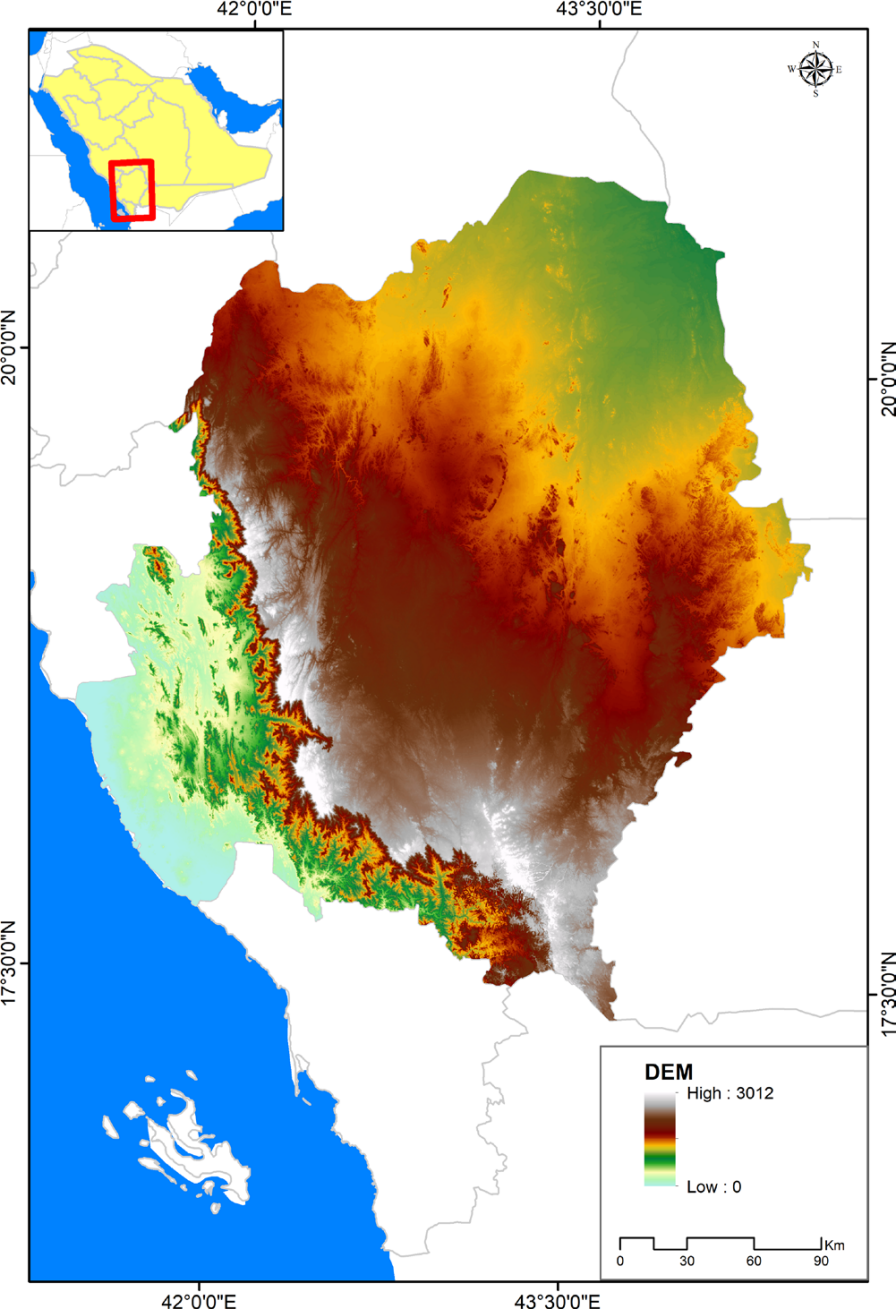
Map of the Aseer region.

This cross-sectional study was conducted in the Aseer Region, a geographically and ecologically diverse administrative province located in the southwestern part of Saudi Arabia. Bordered by Yemen to the south and stretching from the Red Sea coast to the Sarawat mountain range, the region features a wide range of terrains, including coastal plains, high-altitude mountains reaching up to 3000 m, fertile valleys, and arid zones. With a population exceeding 2.3 million, Aseer encompasses both urban centers and rural communities, offering a rich socio-cultural landscape (Fig. 1).

### 2.2. Study population and sampling technique

A total of 100 people were recrui ted using the convenience sampling method. Since our cohort does not include sea-level control subjects, we compared our results with historical reference Hb mass values for sea-level residents. Participants were included if they came from the altitude Aseer Region, were generally healthy with no medical illnesses, and did not take any prescribed medications. Participants were excluded if the initial Hb and HCT were outside the current reference range.

### 2.3. Study procedure and data collection

All participants were provided with an informational video about the procedure and signed informed consent forms. The participants underwent brief interviews to inquire about any medical conditions, drug use, or smoking.

#### Anthropometric measurement

Body weight was measured to the nearest 0.1 kg using a calibrated digital scale, with participants wearing light clothing and no shoes. Height was measured to the nearest 0.1 cm using a wall-mounted stadiometer, with participants standing upright and barefoot. Body mass index (BMI) was calculated using the standard formula:


$$\begin{array}{l} BMI=\frac{Weight\;(kg)}{Height\;{\left(m\right)}^{2}} \end{array}$$

After the initial analysis of the first 50 participants, the study protocol was amended to include a lean body mass (LBM) assessment using DEXA (Lunar iDXA, GE healthcare). BV measurement via CO rebreathing technique followed a previously published and validated protocol ^[6]^ that has also been validated for clinical use.^[7]^ Participants were seated in a recumbent position and an IV line was secured in the dorsum of 1 hand. Venous blood samples were collected and analyzed for Hb (g/dL), HCT (%) and carboxyhemoglobin (%) using a Siemens Rapidpoint 500e analyzer. CO gas (99.9% pure) was administered at a 1 mL/kg dose using the Detalo CO rebreather device (Detalo Performance, Detalo Health, Denmark) with the 4-minute protocol. A waiting period of 6 minutes was allowed before drawing another venous sample, and CO-Hb% was immediately determined on site, with the delta change in CO-Hb% being used to calculate Hb mass. Meanwhile, total BV and PV were derived from Hb and HCT values.

### 2.4. Statistical analysis

Statistical analysis was performed using the Statistical Package for the Social Sciences (SPSS) version 27 (SPSS Inc., Chicago). Descriptive statistics, including mean and standard deviation, were used to summarize the demographic and physical characteristics of the participants, including age, height, weight, and BMI. Frequencies and percentages were used to describe categorical variables, such as gender. Independent-sample *t*-tests were conducted to compare mean differences in blood parameters (such as HCT, Hb concentration, and BVs) between men and women. Mann–Whitney *U* test was used when the data are not normally distributed. Pearson correlation coefficients were calculated to assess the association between various parameters of hematological and BV, while Sperman was used if data was not normally distributed. Statistical significance was established at *P* < .05. Hb mass, total BV, RBCV, and PV were reported as absolute values and normalized to total body weight (BW, kg) and LBM (kg^LBM^). Since an empirical reference range is not available, the obtained Hb mass and BV measurements were compared to recently published predicted reference range values (historical control) ^[8]^ derived as follows: Hb mass (g) = 543.7*height (m) + 3.5*weight (kg) − 547.5 (females) or −349.0 (males), and Hb mass(g) = 166.8*height (m) + 10.8*LBM (kg) − 139.9 (females) or −50.3 (males) when LBM is available.^[8]^

### 2.5. Ethical approval

The study protocol was approved by the Institutional Review Board of King Khalid University (ECM#2023-105) and followed the Declaration of Helsinki. Participants were recruited through directed advertisements targeting university students and clinic visitors.

## 3. Results

### 3.1. Study participant anthropometrics

We excluded 6 participants due to Hb or HCT were outside the reference range. The presented table offers descriptive statistics of key anthropometric variables for the total study population (n = 94) and stratified by sex (male n = 64; female n = 30). The mean age was higher among males (27.8 ± 12.3 years) compared to females (21.5 ± 2.1 years). Males were taller (169.9 ± 6.0 cm) and heavier (81.3 ± 18.4 kg) on average than females (156.7 ± 5.6 cm and 62.6 ± 17.6 kg). The BMI reported for males was higher than females (28.1 ± 5.8 vs 25.3 ± 5.9). LBM was higher among males, averaging 48.8 ± 7.5 kg, compared to females, 32.9 ± 4.4 kg (Table 1).

**Table 1:** Summary of study participant anthropometrics.

Studied variables		Total (n = 94)	Male (n = 64)	Female (n = 30)
Age (yr)	Mean ± SD	25.8 ± 10.6	27.8 ± 12.3	21.5 ± 2.1
	Range	18.0–64.0	18.0–64.0	19.0–29.0
Height	Mean ± SD	165.7 ± 8.5	169.9 ± 6.0	156.7 ± 5.6
	Range	145.0–185.5	158.0–185.0	145.0–170.0
Weight	Mean ± SD	81.3 ± 18.4	81.3 ± 18.4	62.6 ± 17.6
	Range	41–130	41.0–130.0	41.1–111.0
BMI	Mean ± SD	25.3 ± 1.1	28.1 ± 5.8	25.3 ± 5.9
	Range	15.2–43.4	17.9–39.8	15.2–43.4
LBM	Mean ± SD	48.8 ± 7.5	48.8 ± 7.5	32.9 ± 4.4
	Range	32.13–62.4	26.0–40.3	32.2–62.4

Age (y r), height (cm), weight (kg), body mass index (BMI; kg/m^2^), and lean body mass (LBM; kg) in male, female, and all study participants.

BMI = body mass index, LBM = lean body mass.

### 3.2. Total blood, red blood cell, and PV measurements

Hb mass was higher in males (922.8 ± 195.0 g) compared to females (594.9 ± 130.4 g; *P* < .0001). Additionally, males had a higher mean Hb mass per kg (11.7 ± 3.1 g/kg vs 9.9 ± 2.4 g/kg in females, *P* = .007). However, when Hb mass was expressed per LBM, there was no difference between males and females (18.5 ± 3.0 g/kg^LBM^ compared to 17.6 ± 3.8 g/kg^LBM^, *P* = .375) (Table 2).

**Table 2:** Hematological variables (Mean ± SD) by sex in study participants.

Blood parameter	Female (n = 30)	Female* (n = 263)	Male (n = 64)	Male* (n = 319)	*P*
Hematocrit (%)	41.4 ± 3.7	40.3 ± 2.7	48.6 ± 3.7	43.7 ± 2.9	.0001
Hb (g/dL)	14.1 ± 1.2	13.4 ± 0.9	16.5 ± 1.3	14.7 ± 1.1	.0001
Hb mass (g)	594.9 ± 130.4	594.9 ± 91.2	922.8 ± 195.0	901 ± 123	.0001
Hb mass (g), predicted^BW^	523 ± 85		860 ± 84		.0001
Hb mass (g), predicted^LBM^	476.8 ± 54.8		759.8 ± 87.2		.0001
Hb mass/kg^BW^ (g/kg)	9.9 ± 2.4	8.9 ± 1.4	11.7 ± 3.1	11.5 ± 1.5	.007
Hb mass/kg^LBM^ (g/kg)	17.6 ± 3.8	14.0 ± 1.5	18.5 ± 3.0	14.9 ± 1.6	.375
Blood volume (mL)	4231.0 ± 881.0	4682.0 ± 738.0	5625.0 ± 1209.0	6279.0 ± 829.0	.0001
Blood volume (mL)^BW^	70.3 ± 15.3	70.3 ± 11.3	72.3 ± 22.2	80.3 ± 10.8	.658
Blood volume (mL)^LBM^	126.8 ± 23.7	110.9 ± 12.5	110.9 ± 15.3	105.3 ± 11.2	.007
Red blood cell volume (mL)	1749.8 ± 37.8	1823 ± 299	2721.2 ± 57.9	2708 ± 411	.0001
Red blood cell volume (mL)^BW^	29.2 ± 7.1	27.4 ± 4.8	34.6 ± 8.8	34.6 ± 5.0	.004
Red blood cell volume (mL)^LBM^	51.8 ± 11.1	43.2 ± 5.3	54.3 ± 8.8	45.1 ± 5.5	.39
Plasma volume (mL)	2480 ± 552	2861 ± 480	2903 ± 717	3570 ± 510	.005
Plasma volume (mL)^BW^	41.1 ± 9.1	43.0 ± 7.2	37.3 ± 13.3	45.7 ± 6.9	.155
Plasma volume (mL)^LBM^	75.0 ± 14.2	67.7 ± 8.6	56.6 ± 8.2	60.2 ± 7.7	.0001

Hematocrit (%), Hb (g/dL), Hb mass, and blood volumes (BVs) in female and male study participants and the corresponding reference population for comparison. *P*-values indicate differences between sexes of the study cohort.

BVs = blood volumes, BW = body weight, LBM = lean body mass, SD = standard deviation .

* Historical reference.

†For LBM-derived measurements, female (n = 21), male (n = 28).

Males had higher absolute values for BV, RBCV, and PV. BV was 5625.0 ± 1209.0 mL in males compared to 4231.0 ± 881.0 mL in females (*P* < .0001). However, BV was not different between sexes when expressed per measured body weight (72.3 ± 22.2 vs 70.3 ± 15.3 mL/kg, respectively, *P* = .658). Furthermore, when expressed per LBM, BV was higher in females (126.8 ± 23.7 vs 110.9 ± 15.3 mL/kg^LBM^, *P* = .007). The higher BV^LBM^ in females was related to a higher PV (75.0 ± 14.2 vs 56.6 ± 8.2 mL/kg^LBM^ *P* < .001).

As stated above, RBCV expressed as absolute values was higher in males (2721 ± 57.9 mL) than in females (1749.8 ± 37.8 mL, *P* = .0001), and also when expressed per body weight (34.6 ± 8.8 vs 29.2 ± 7.1 mL/kg, *P* = .004). However, the difference disappeared when RBCV was expressed per LBM (54.3 ± 8.8 vs 51.8 ± 11.1 mL/kg^LBM^, *P* = .39).

Likewise, absolute PV was higher in men than in women (2903 ± 717 vs 2480 ± 552 mL, *P* = .005), but no difference remained when PV was expressed per body weight (37.3 ± 13.3 vs 41.1 ± 9.1 mL/kg, *P* = .155). When expressed per LBM, females displayed higher PV (56.6 mL ± 8.2 mL/kg^LBM^ in males vs 75.0 ± 14.2 mL/kg^LBM^ in females; *P* < .0001) (Table 2).

### 3.3. Comparison of Hb mass with sea-level reference values

For the total weight-based prediction of Hb mass, the altitude cohort showed higher Hb mass in both males (922.8 ± 195.0 vs 860 ± 84 g, *P* = .01) and females (594.9 ± 130.4 vs 523 ± 84.6 g, *P* = .014). When using the prediction based on LBM, higher Hb mass was again observed in both altitude males (922.8 ± 195.0 vs 759.8 ± 87.2 g/kg^LBM^) and females (594.9 ± 130.4 vs 476.9 ± 54.8 g/kg^LBM^) (*P* < .0001 for both) (Table 2).

### 3.4. Correlation of Hb and HCT with Hb mass and intravascular volumes

Total Hb mass displayed a non-significant correlation with Hb and HCT in both genders. Meanwhile, Hb mass^LBM^ showed significant positive correlations with both Hb and HCT in both sexes, with *P *< .05. Red BV relative to LBM (RBCV^LBM^) was also positively correlated with both Hb and HCT for both genders. In contrast, Hb mass relative to body weight (Hb mass^BW^) demonstrated mixed results. For females, there was a significant positive correlation with Hb and HCT, though correlations with other parameters were weaker or not significant. For males, correlations with Hb and HCT were not significant. Finally, PV correlations revealed significant negative correlation, consistent with expectations. Specifically, both males and females showed significant negative correlations of Hb and HCT with PV and PV^BW^ (Table 3).

**Table 3:** Pearson correlations of Hb and HCT with Hb mass and intravascular volumes.

Studied parameters	Females				Males			
	Hb (g/dL)		Hematocrit (%)		Hb (g/dL)		Hematocrit (%)	
	*r*	*P*	*r*	*P*	*r*	*P*	*r*	*P*
Hb mass (g)	0.307	.099	0.321	.083	0.196	.121	0.154	.224
HB mass^BW^	0.467	.009	0.446	.014	−0.096	.448	−0.077	.544
Hb mass^LBM^	0.487	.025	0.481	.027	0.532	.004	0.527	.004
Red blood cell volume	0.311	.095	0.329	.076	0.18	.154	0.163	.199
Red blood volume^BW^	0.476	.008	0.458	.011	−0.109	.393	−0.069	.586
Red blood volume^LBM^	0.489	.025	0.486	.026	0.529	.004	0.527	.004
Plasma volume	−0.386	.035	−0.371	.044	−0.454	<.001	−0.491	<.001
Plasma volume^BW^	−0.148	.436	−0.172	.363	−0.539	<.001	−0.530	.001
Plasma volume^LBM^	−0.205	.372	−0.212	.357	−0.327	−.09	0.331	.085

BW = body weight, Hb = hemoglobin, LBM = lean body mass, *r* = correlation coefficient; *P* = significance.

## 4. Discussion

In clinical practice, Hb and HCT measures are often used as surrogates for intravascular volumes, as the traditional methods used to quantify these volumes are not considered applicable in most clinical settings. In the present study, we made use of a CO rebreathing technique, which is proven safe and easy to use in the clinical setting.^[9–11]^ Our findings demonstrate that in a healthy population living at moderate altitude, neither Hb or HCT correlate well with Hb mass or intravascular volumes. Furthermore, our work also corroborates a previous finding that LBM is of greater value than overall body weight for the interpretation of these measurements.^[8]^

Our data show that Hb mass, RBCV, and BV are higher in males compared to females when expressed as absolute values or normalized to total body weight. However, in accordance with previous findings,^[8]^ when Hb mass and intravascular volumes were expressed relative to LBM, the observed sex-related differences in Hb mass and RCBV greatly diminished. Indeed, females displayed a higher total BV^LBM^, which was explained by a higher PV^LBM^. These findings demonstrate that LBM is important to obtaining a clinically reliable and useful BVs measurements. Indeed, as fat tissue is hypovascular, normalization to LBM is expected to provide more clinically valid measurements, especially in people who are obese. Therefore, it is recommended that LBM be used for the reporting of BV values, particularly when obesity is involved.^[12,13]^

To explore the effect of moderate altitude on Hb mass and intravascular volumes, we compared the results of our cohort with reference values for individuals who live at sea level.^[8]^ Our results showed a predictable change in BV consistent with high-altitude adaptation, with Hb mass increased by 13.5% in females (mean 594.9 ± 130 g compared to predicted 523.5 ± 84.7 g) and by 7% in males (922.8 ± 195.0 g compared to 859.6 ± 84.2 g). Therefore, in our cohort, the Hb mass is higher than reported at sea level, but lower than reported in smaller groups at higher altitude (3800 m)^[14]^ In particular, when values were expressed per kilogram of body mass, no effect of altitude could be found. However, when expressed relative to LBM, our cohort showed a positive altitude effect on Hb mass for both females (17.6 ± 3.8 g/kg^LBM^ compared to 14.0 ± 1.5 g/kg^LBM^) and males (18.5 ± 3.0 g/kg^LBM^ compared to 14.9 ± 1.6 g/kg^LBM^). The reason for this discrepancy is that the present cohort had a higher body mass compared to the reference population, and the higher body mass was driven by a higher fat mass. Likewise, for RBCV^LBM^, males and females both displayed elevated values compared to the sea-level reference population. For PV^LBM^, our altitude population displayed low values in males relative to the reference population, but also elevated values in females. The latter may be related to the small size of our cohort with available LBM (21 female subjects). It should also be noted that the reference values were obtained from a Caucasian cohort and that a local Saudi Arabian sea-level control would strictly be preferred.

Together, the results obtained indicate that in males, and to lesser degree in females, the increased Hb at high-altitude can be attributed to both higher RBCV and reduced PV. By extension, when diagnosing polycythemia and anemia from Hb values at high-altitudes, determinations should not be based exclusively on the impact of RBCV on Hb but should take into account the reduction in PV.

Although physiological adaptation to high altitude and associated changes in intravascular volume status may explain the poor correlation of Hb and HCT values with true red cell mass, pathological conditions can further exacerbate this discrepancy. For example, anemia in patients with chronic kidney disease is to a great extent the result of PV expansion, i.e. dilutional anemia. Using Hb as a diagnostic tool for anemia in such cases could result in overtreatment with erythropoietin, a drug known to be associated with increased mortality.^[2]^ Indeed, in patients with heart failure, a blood-volume-guided treatment for anemia or diuresis is reportedly associated with better outcomes.^[15,16]^

The evaluation of polycythemia at high altitudes is complicated by the prevalence of altitude-associated polycythemia. Furthermore, it is known that expansion of both PV and RBCV can occur with polycythemia vera and thus present normal Hb and HCT, so-called masked polycythemia.^[17]^ To account for this occurrence, the Hb and HCT thresholds for considering polycythemia have been lowered to 16 g/dL and 48% for women and 16.5 g/dL and 49% for men.^[18]^ These cutoff values overlap considerably with normal ranges at high altitudes, underscoring the need for better screening methods to select individuals for polycythemia evaluation at high altitudes. The RBC (Hb) mass >25% compared to the reference is one of the diagnostic criteria for polycythemia vera and relies on a validated reference range.^[19]^ Hence, for high-altitude populations, a robust reference range needs to be established; our cohort is an initial attempt towards this effort. In particular, *JAK2* mutation is invariably present in all patients with polycythemia vera, and observation of the characteristic bone marrow morphology of pan-myelosis would obviate the need for measurement of measurement of red cell mass^[1]^; however, such an invasive test is likely not justified as a patient screening tool. Furthermore, Hb mass quantification is valuable in differentiating essential thrombocytosis from polycythemia vera with normal Hb and HCT, which distinction is a very important therapeutic consideration.^[20,21]^

How else can we deal with the shortcomings of using Hb and HCT for diagnosing anemia and polycythemia at high altitudes? The World Health Organization has acknowledged the impact of altitude on Hb and suggested a correction factor when defining anemia at higher altitudes.^[5]^ However, subsequent studies have questioned the validity and generalizability of this approach. It has been demonstrated that Hb elevation is not universal and can be influenced by factors such as race and region,^[22]^ with genetic adaptations also involved in population responses to high altitude.^[23]^ The WHO-suggested adjustments are unlikely to overcome the fundamentally poor correlation of Hb with actual Hb mass, never mind its further exacerbation by the known effects of altitude on Hb mass and PV in both short and long terms. More studies are needed to quantify the degree to which changes of Hb mass or PV contribute to measured Hb and HCT at a given altitude before these hematological parameters can reliably be used. Until that day, direct measurement provides the most reliable indicator of true changes in Hb mass and intravascular BVs.

### 4.1. Strengths and limitations

This study is limited by the small number of participants, especially the fraction having available LBM measurements, and by the absence of a local sea-level control cohort. Establishing a validated altitude and population-based reference range is needed before routinely using CO rebreathing for this purpose in clinical practice.

## 5. Conclusions

When corrected for LBM, gender differences in Hb mass and RBCV diminish greatly. LBM is probably needed to reliably interpret Hb mass and intravascular volume status as measured by CO rebreathing. Moderate altitude residents have elevated Hb mass compared to sea-level reference values. Local sea-level controls are needed to reliably establish true altitude-associated changes in Hb mass. Ultimately, assessment of Hb mass by CO rebreathing could help select patients for further evaluation of polycythemia and in those with masked polycythemia (increased Hb mass with normal Hb and HCT). However, the establishment of a validated population- and altitude-based reference range is an important requisite for applying this method in clinical practice.

## Acknowledgments

The authors thank all the participants of this study for their commitment and the staff at the radiology and outpatient department of King Khalid University Hospital for facilitating this project. The authors also extend their appreciation to the Ministry of Education in KSA for funding this research work through project number KKU-IFP2-H-30.

## Author contributions

**Conceptualization:** Husain Alkhaldy, Mohammed Alshehri.

**Investigation:** Husain Alkhaldy, Mahdi S. Al Amri, Meshal M. Alqahtani, Yusra D. Alasmari.

**Methodology:** Husain Alkhaldy, Meshal M. Alqahtani, Yusra D. Alasmari.

**Software:** Ramy Mohamed Ghazy.

**Supervision:** Husain Alkhaldy, Ramy Mohamed Ghazy, Mohammed Alshehri.

**Validation:** Husain Alkhaldy, Yusra D. Alasmari.

**Writing – original draft:** Husain Alkhaldy, Mohammed Alshehri, Ramy Mohamed Ghazy.

**Writing – review & editing:** Husain Alkhaldy, Mohammed Alshehri, Ramy Mohamed Ghazy.
